# Approximating the stabilization of cellular metabolism by compartmentalization

**DOI:** 10.1007/s12064-016-0225-y

**Published:** 2016-04-05

**Authors:** Lisa Fürtauer, Thomas Nägele

**Affiliations:** Department of Ecogenomics and Systems Biology, University of Vienna, Althanstr. 14, 1090 Vienna, Austria; Vienna Metabolomics Center (VIME), University of Vienna, Althanstr. 14, 1090 Vienna, Austria

**Keywords:** Stability, Subcellular network, Eigenvalue, Jacobian matrix, Compartmentalization, Systems biology

## Abstract

Biochemical regulation in compartmentalized metabolic networks is highly complex and non-intuitive. This is particularly true for cells of higher plants showing one of the most compartmentalized cellular structures across all kingdoms of life. The interpretation and testable hypothesis generation from experimental data on such complex systems is a challenging step in biological research and biotechnological applications. While it is known that subcellular compartments provide defined reaction spaces within a cell allowing for the tight coordination of complex biochemical reaction sequences, its role in the coordination of metabolic signals during metabolic reprogramming due to environmental fluctuations is less clear. In the present study, we numerically analysed the effects of environmental fluctuations in a subcellular metabolic network with regard to the stability of an experimentally observed steady state in the genetic model plant *Arabidopsis thaliana*. Applying a method for kinetic parameter normalization, several millions of probable enzyme kinetic parameter constellations were simulated and evaluated with regard to the stability information of the metabolic homeostasis. Information about the stability of the metabolic steady state was derived from real parts of eigenvalues of Jacobian matrices. Our results provide evidence for a differential stabilizing contribution of different subcellular compartments. We could identify stabilizing and destabilizing network components which we could classify according to their subcellular localization. The findings prove that a highly dynamic interplay between intracellular compartments is preliminary for an efficient stabilization of a metabolic homeostasis after environmental perturbation. Further, our results provide evidence that feedback-inhibition originating from the cytosol and plastid seem to stabilize the sucrose homeostasis more efficiently than vacuolar control. In summary, our results indicate stabilizing and destabilizing network components in context of their subcellular organization.

**Electronic supplementary material:**

The online version of this article (doi:10.1007/s12064-016-0225-y) contains supplementary material, which is available to authorized users.

## Introduction

A characteristic feature of eukaryotic cells is a compartmentalized subcellular structure resulting in numerous cellular reaction environments. Due to this compartmentalization, various biochemical reaction conditions are established in a cell enabling highly coordinated and tightly regulated reactions and reaction sequences. Particularly, such highly compartmentalized subcellular structures are a characteristic feature of plant cells (Lunn 2007). The first steps of converting light to chemically usable energy, i.e. the reaction sequence of photosynthetic carbon fixation and the central carbohydrate metabolism, are tightly linked and regulated which allows plants to efficiently respond and acclimate to environmental fluctuations (Yamori et al. 2014). The subcellular compartments chloroplast, cytosol and vacuole are connected by various transporters and channels which enable the regulated exchange of metabolic compounds across biological membrane systems (Linka and Weber 2010). Finally, this results in a highly complex metabolic network which has been analysed by various approaches of mathematical modelling (see e.g. Grafahrend-Belau et al. 2013; Morgan and Rhodes 2002; Nägele and Weckwerth 2013; Pokhilko et al. 2014).

As sessile organisms, plants have to cope with fast and fluctuating changes in environmental conditions, e.g. a sudden increase/decrease in light intensity or temperature. To prevent a disbalance of primary and secondary photosynthetic reactions, which could easily cause the generation of reactive oxygen species (ROS) and the irreversible damage of membrane systems and cells, a new and stable metabolic homeostasis has to be established within a relatively short time period. In biochemical networks, the metabolic homeostasis results from a concerted orchestration of enzymatic activities which determine metabolic fluxes, i.e. rates of metabolic interconversion and membrane transport. The coordinate reprogramming of a metabolic homeostasis, which finally results in a new acclimated stable homeostasis, is a complex process comprising numerous enzymatic reactions and regulatory molecular circuits. Decades ago it was outlined that by linear approximation of enzymatic reaction chains, i.e. the assumption that enzyme velocities linearly depend on substrate concentrations, analytical solutions can be obtained for metabolite concentrations and metabolic fluxes at steady state conditions (Heinrich and Rapoport 1974). Defining the cardinal terms control strength, control matrix and effector strength, Heinrich and Rapoport defined the dependence of metabolic flux and metabolite concentrations on enzyme kinetic properties as well as on effector concentrations. Together with the independently developed work of Kacser and Burns (Kacser and Burns 1973) this provided the basic concept for the research field of Metabolic Control Analysis as it was summarized more than two decades later (Kacser et al. 1995).

The stability of an observed metabolic steady-state can be numerically characterized by the eigenvalues of the related Jacobian matrix (Reznik and Segrè 2010). One major obstacle in determination of the Jacobian is the lack of experimental data on enzyme kinetic parameters, e.g. the maximum velocity, *v*_max_, or the substrate affinity, *K*_M_. To overcome this limitation, Steuer and co-workers have developed the approach of Structural Kinetic Modelling, SKM, in which normalized enzyme kinetic parameters replace conventional parameters (Steuer et al. 2006). This approach has been proven to be suitable for identification of stability and robustness of metabolic states (Grimbs et al. 2007), but also for specific network structures like metabolic cycles (Reznik and Segrè 2010). In a previous approach we have applied the SKM approach to determine stability properties of a highly simplified reaction core of the central carbohydrate metabolism in plant leaves (Henkel et al. 2011). While we could already provide evidence for a differential stabilization effect of feedback inhibition originating from different metabolic sites in a small metabolic cycle belonging to the central carbohydrate metabolism (Henkel et al. 2011), we did not consider a possible effect of subcellular compartmentalization. Here, we enlarge our previous approach aiming at a comprehensive understanding of how a subcellular allocation of central metabolic compounds affects the stability properties of a metabolic homeostasis. Finally, our approach intends to shed light on one of the most characteristic features of eukaryotic cells in context of a successful acclimation strategy to a fluctuating environment.

## Biological model preliminaries

**Fig. 1:**
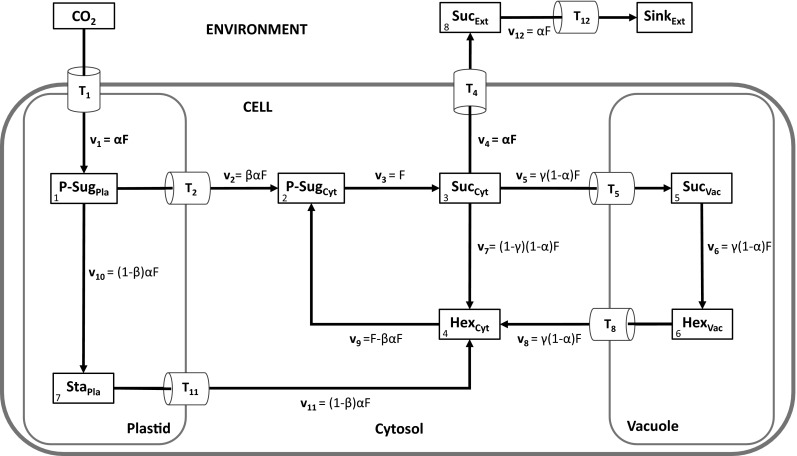
Schematic model of the central subcellular carbohydrate metabolism in plant leaf mesophyll cells. The assimilation of CO_2_ supplies the pool of plastidial sugar phosphates (P-Sug_Pla_) which is the first metabolic substrate for all other reactions. Metabolites are written in *boxes*, transport systems are indicated by *T*_*j*_ and enzyme-driven interconverting reactions by *v*_*j*_. Steady state fluxes for all reactions are additionally shown (e.g. *αF*). *Pla* plastidial, *Cyt* cytosolic, *Vac* vacuolar, *Ext* extracellular, *P-Sug* sugar-phosphates, *Sta* starch, *Suc* sucrose, *Hex* hexoses

Based on approaches of mathematical modelling in previous publications (Nägele et al. 2010; Nägele and Heyer 2013; Nägele et al. 2012), we derived a simplified, but still representative, metabolic model of the central carbohydrate metabolism in leaf mesophyll cells (Fig. 1). The model comprised the three intracellular compartments plastid, cytosol and vacuole, as well as an extracellular environment. Transporters (*T*_*j*_) were interpreted as a pool of all involved membrane transporters, even though some of these transporters might still have to be characterized (Lunn 2007; Linka and Weber 2010; Bräutigam and Weber 2011). The influx of the system, *T*_1_ and *v*_1_, represented the complete process of CO_2_ transport and fixation resulting in the pool of plastidial sugar phosphates (P-Sug_Pla_). Environmental fluctuations α, e.g. occurring due to sudden changes in light intensity or temperature, directly affected the flux *F* of *v*_1_ as a constant scalar (*v*_1_ = *αF*). The pool of plastidial sugar phosphates was substrate for two reactions: v_2_, the export to the cytosol (P-Sug_Cyt_), catalysed by membrane transporters, and *v*_10_, representing the intraplastidial conversion to starch (Sta_Pla_). Cytosolic sucrose, Suc_Cyt_, was the substrate for the export reaction via *T*_4_ supplying the pool of extracellular sucrose, Suc_Ext_. Reaction *v*_5_ indicated the transport of sucrose from the cytosol (Suc_Cyt_) to the vacuole (Suc_Vac_). In both compartments, hydrolytic cleavage of sucrose via invertase yielded free hexoses [*v*_6_ (Hex_Vac_) and *v*_7_ (Hex_Cyt_)]. Vacuolar hexose transport to the cytosol was described via transport process *T*_8_ resulting in a circular structure. Degradation of plastidial starch (Sta_Pla_) to hexose equivalents and their transport to the cytosol was described by transport *T*_11_ and reaction *v*_11_. Reaction *v*_9_ described the phosphorylation of cytosolic hexoses via hexokinase activity (Claeyssen and Rivoal 2007), supplying the pool of sugar phosphates (P-Sug_Cyt_). Finally, reaction *v*_12_ which was coupled to transport *T*_12_, described the transport of apoplastic sucrose, Suc_Ext_, to sink organs (Sink_Ext_).

## Mathematical model preliminaries

The model comprised 12 reactions (*v*_*j*_; *j* = 1,…,*r* here: *r* = 12), steady state concentrations of different metabolites (*c*_0,*i*_*i* = 1,…,*m* here: *m* = 8) and their fluxes *ν*_*j*_(*c*_0,*i*_). Experimentally determined steady state concentrations of metabolites, which are provided in the supplements (Supplementary Table S1), were derived from previous studies (Nägele and Heyer 2013; Nägele et al. 2012). Steady state concentrations of extracellular sucrose (Suc_Ext_), which were not experimentally determined in these studies, were set to a low (0.01 mM), medium (15 mM) and a high (45 mM) concentration when compared to the cytosolic sucrose concentration (15 mM). With these settings, three possible scenarios were simulated: (1) a sucrose concentration gradient from the intracellular to the extracellular space (Suc_Ext_ low), (2) no sucrose gradient (Suc_Ext_ medium), and (3) a sucrose concentration gradient from the extracellular to the intracellular space (Suc_Ext_ high). To define a steady state equilibrium, the input flux *v*_1_ and output flux *v*_4_ were set to an equal value (*αF*). The flux *F* was set to be constant (*F* = 1). For simulation of random environmental fluctuations and the response of the system, alpha (α) and the proportion characters beta (*β*) and gamma (*γ*), were randomly varied in the interval (0; 1). While *β* quantified the relative proportion of carbon flux from chloroplast to cytosol, *γ* quantified the proportion of carbon flux from cytosol to vacuole. Based on the SKM approach (Steuer et al. 2006), the Jacobian matrix **J** was defined as the product of matrices **Λ** and **θ** (Eq. 1):

1 $$J: = \varLambda \theta$$

Here, **Λ** is the stoichiometric matrix (**N**) of the considered metabolic system, normalized to steady-state fluxes *ν*(*c*_0_) and metabolite concentrations *c*_0,*i*_ (Eq. 2):

2 $$\varLambda_{i,j} : = N_{i,j} \frac{{v_{j} (c_{0,i} )}}{{c_{0,i} }}$$

Entries of the matrix **θ** represent normalized elasticities, i.e. the degree of saturation of a normalized flux µ with regard to the normalized substrate concentration *x* (Eq. 3):

3 $$\theta : = \frac{{{\text{d}}\mu }}{{{\text{d}}x}} = \frac{{{\text{d}}\frac{v(c)}{{v(c_{0} )}}}}{{{\text{d}}\frac{c(t)}{{c_{0} }}}}$$

A non-zero element $\theta_{j,i}^{{}}$ in matrix **θ** indicated the involvement of a metabolite as a substrate, as an activating or inhibiting compound. In case of activating compounds, elasticities in **θ** were defined within the interval (0; 1], while for inhibitory compounds the interval was (−1; 0). A detailed explanation of these entries in context of enzyme kinetics, such as Michaelis–Menten kinetics, was provided previously (Reznik and Segrè 2010).

A detailed and explicit formulation of the **Λ** and **θ** matrices derived in the present study is provided in the Supplements (Supplementary files S2 and S3) while a short and very fundamental explanation of eigenvalues in context of a differential equation is provided in the following paragraph.

Eigenvalues of the Jacobian **J** characterize a metabolic steady state with regard to its stability. The steady state concentration *c*_0,i_ of a metabolite c_i_, is related to the time-dependent concentration *c*_*i*_(*t*) by a fluctuation term $\tau_{i} (t)$ (Eq. 4):

4 $$c_{i} (t) = c_{0,i} + \tau_{i} (t)$$

The first element of a Taylor expansion reveals the linearization around the considered steady state (Eq. 5):

5 $$\frac{{{\text{d}}\tau_{i} \left( t \right)}}{{{\text{d}}t}} = \mathop \sum \limits_{p = 1}^{m} J_{i,p} \tau_{p} \left( t \right)$$

Integration yields the general solution (Eq. 6):

6 $$\tau_{i} \left( t \right) = \mathop \sum \limits_{p = 1}^{m} C_{i,p} e^{{\lambda_{p} t}}$$

here, **C** represents constants which depend on the initial fluctuation conditions. The complex number *λ*_*i*_ represents the eigenvalues of the Jacobian matrix **J** (Eq. 7):

7 $$det\left( {\varvec{J} - \lambda \varvec{I}_{\varvec{m}} } \right) = 0$$

**I**_**m**_ represents the unit matrix, and Eq. 7 is known as the characteristic equation.

Hence, the eigenvalue *λ*_*i*_ characterizes the solution of Eq. 6: for re(*λ*_*i*_) < 0, the fluctuation term $\tau_{i} \left( t \right)$ decays exponentially (stability), while re(*λ*_*i*_) > 0 results in an exponential increase (instability). While the imaginary part of *λ*_*i*_, im(*λ*_*i*_), is necessary to exactly classify the local dynamics and to differentiate a node from a focus or a saddle (Steuer 2007), the present study only accounted for the real parts of *λ*_*i*_ which is sufficient to differentiate stable from instable solutions.

Stability characteristics of steady states were evaluated by the maximum real part of the eigenvalue (*λ*_max_). To simulate different activation and inhibition scenarios, the **θ** matrix was adapted accordingly. In general, for an activation, the corresponding $\theta_{j,i}^{{}}$ value was set to 1 to obtain a maximal perturbation. Exceptions from this are directly indicated in the text or in the figures. Inhibition scenarios were simulated with a weak ($\theta_{j,i}^{{}}$ = −0.01) and strong ($\theta_{j,i}^{{}}$ = −0.99) effect. For all models, 10^6^ iterations were calculated to reduce the probability of numerical coincidences. For reactions *v*_2_ to *v*_12_, the enzyme-substrate interaction was described by entries in the elasticity matrix **θ** indicated by the subscript index (first index number, j: reaction; second index number, i: metabolite pool). Elasticities were chosen randomly in the interval of (0; 1) for every iteration process to reveal the system behaviour over all 10^6^ iterations. The source code which was applied for calculations is provided in the supplements (Supplementary file S5).

## Subcellular stability analysis in context of environmental perturbation

**Fig. 2:**
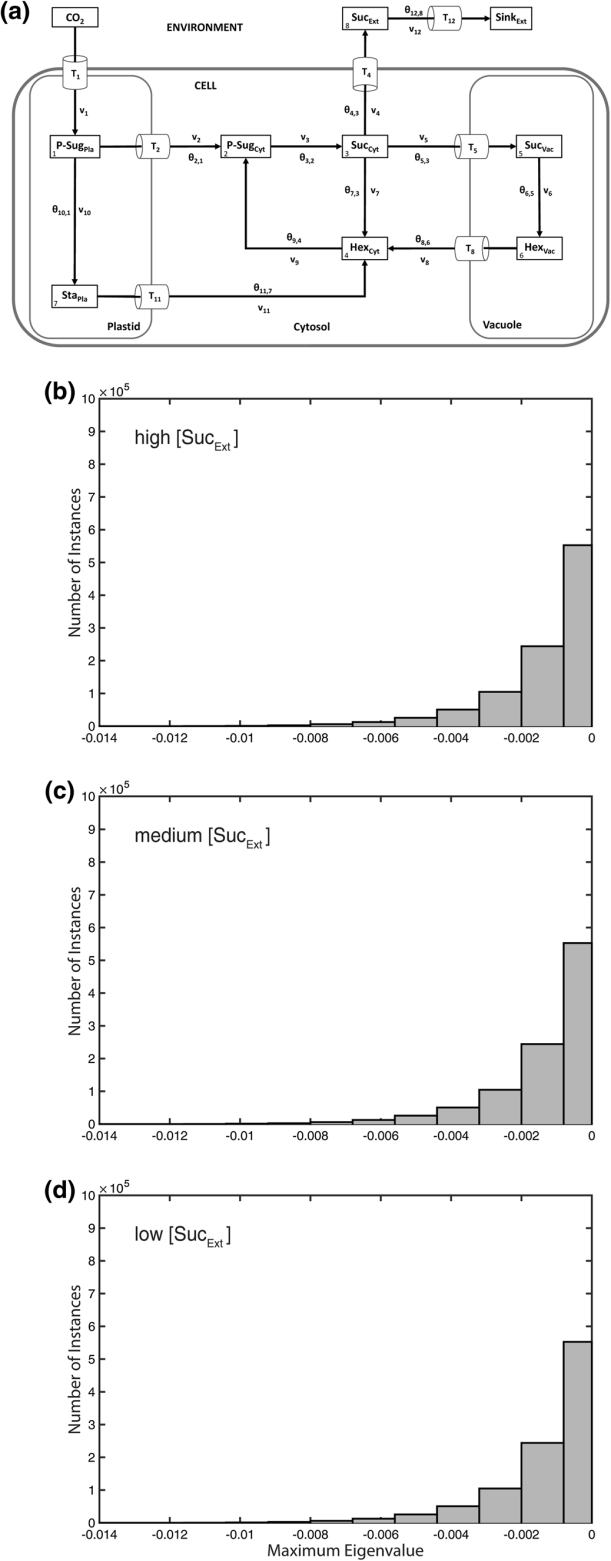
Model structure and histograms of maximum eigenvalue real parts without activation and inhibition. Calculations for the shown model configuration (**a**) were performed 10^6^ times for high (**b**), medium (**c**) and low (**d**) concentration of extracellular/apoplastic sucrose concentrations. Further detailed information, e.g. about maximum values of the eigenvalue real part distribution, is provided in Supplementary Table S4

Simulation of 10^6^ variations, i.e. implicit normalized parameter sets, in the **θ** matrix without any metabolic activation or inhibition yielded only stable systems, irrespective of a variation in external sucrose concentration (Fig. 2a–d). Additionally, in the three Suc_Ext_ concentration scenarios, no differences between resulting *λ*_max_ values were observed (Fig. 2b–d). Conclusively, when steps of metabolic activation and inhibition were absent, a variation of external sucrose levels did not affect the stability properties of the considered intracellular metabolic system.

**Fig. 3:**
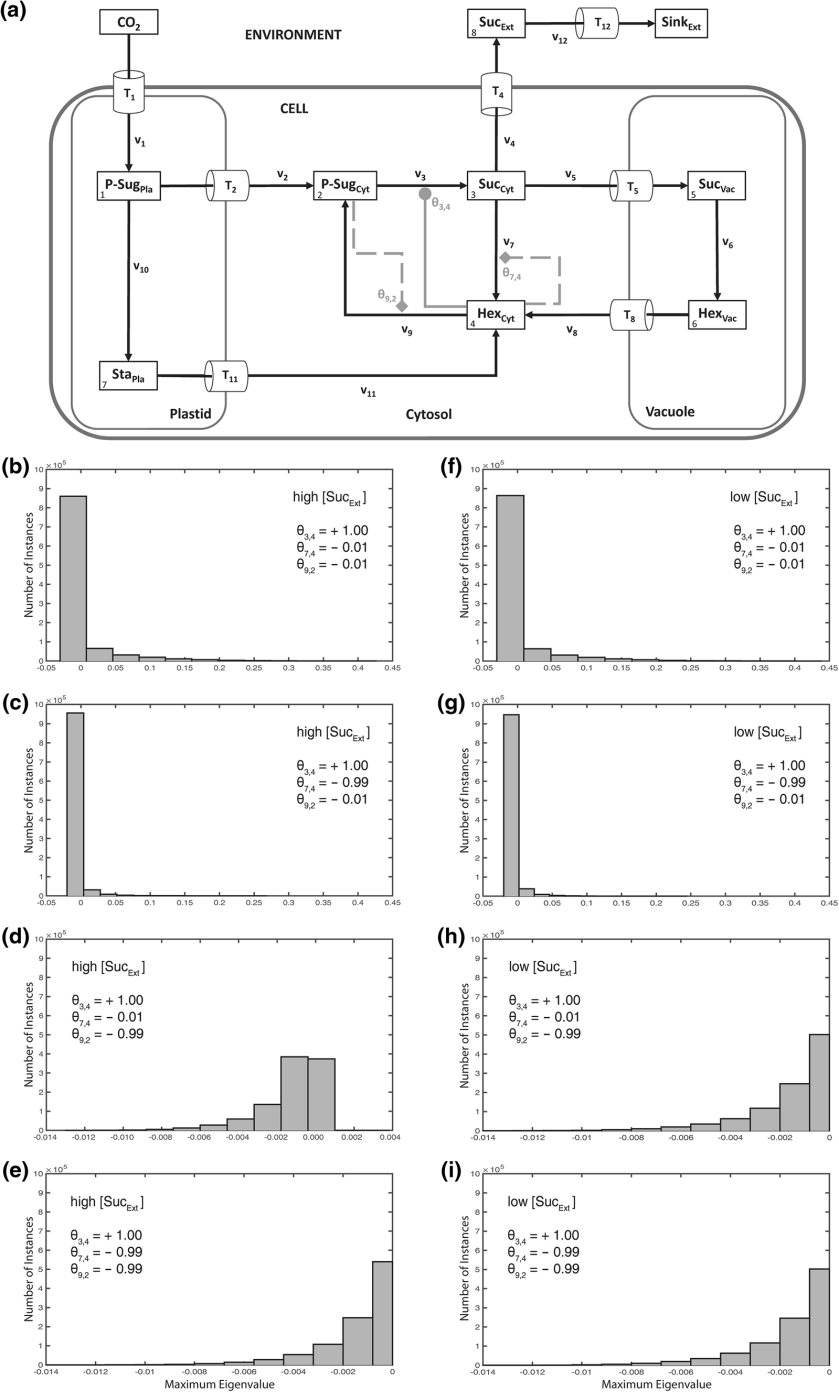
Model structure and histograms of maximum eigenvalue real parts with cytosolic activation and inhibition. Calculations for the shown model configuration (**a**) were performed 10^6^ times for high (**b**–**e**), and low (**f**–**i**) concentration of extracellular/apoplastic sucrose concentrations. Steps of metabolic activation are indicated by *grey filled circles*, steps of inhibition are indicated by *grey filled diamonds* and *dashed lines*. Particular settings in the ***θ*** matrix are indicated within the *single histograms*. Further detailed information, e.g. about maximum values of the eigenvalue real part distribution, is provided in Supplementary Table S4

In the next step, instances of metabolic activation and feedback-inhibition were incorporated into the model, belonging to the central cytosolic carbohydrate metabolism as described previously in a simplified model structure (Henkel et al. 2011). In this model structure, cytosolic hexoses feedforward-activated the biosynthesis of sucrose and feedback-inhibited the cytosolic cleavage of sucrose (Fig. 3a). Additionally, cytosolic sugar phosphates feedback-inhibited the phosphorylation of free cytosolic hexoses. In contrast to the previous observation without any regulatory instance, stability properties were now observed to depend on external sucrose concentrations (Fig. 3b–i). While no difference in system stability was observed as long as feedback-inhibition of cytosolic sugar phosphates was set to be weak (*θ*_9,2_ = −0.01; Fig. 3b, c, f, g), this pattern changed with a strong feedback of cytosolic sugar phosphates and a weak feedback-inhibition of cytosolic hexoses (*θ*_7,4_ = −0.01; Fig. 3d, h): in this constitution, high external sucrose concentrations resulted in instabilities (Fig. 3d). Instabilities, i.e. positive eigenvalue real parts, disappeared again when both feedback-inhibitions, i.e. *θ*_9,2_ and *θ*_7,4_, were defined to be strong (Fig. 3e, i). These stability characteristics were also observed when the steady state flux was divided in half (*F* = 0.5) or doubled (*F* = 2; see Supplementary Figure S6).

**Fig. 4:**
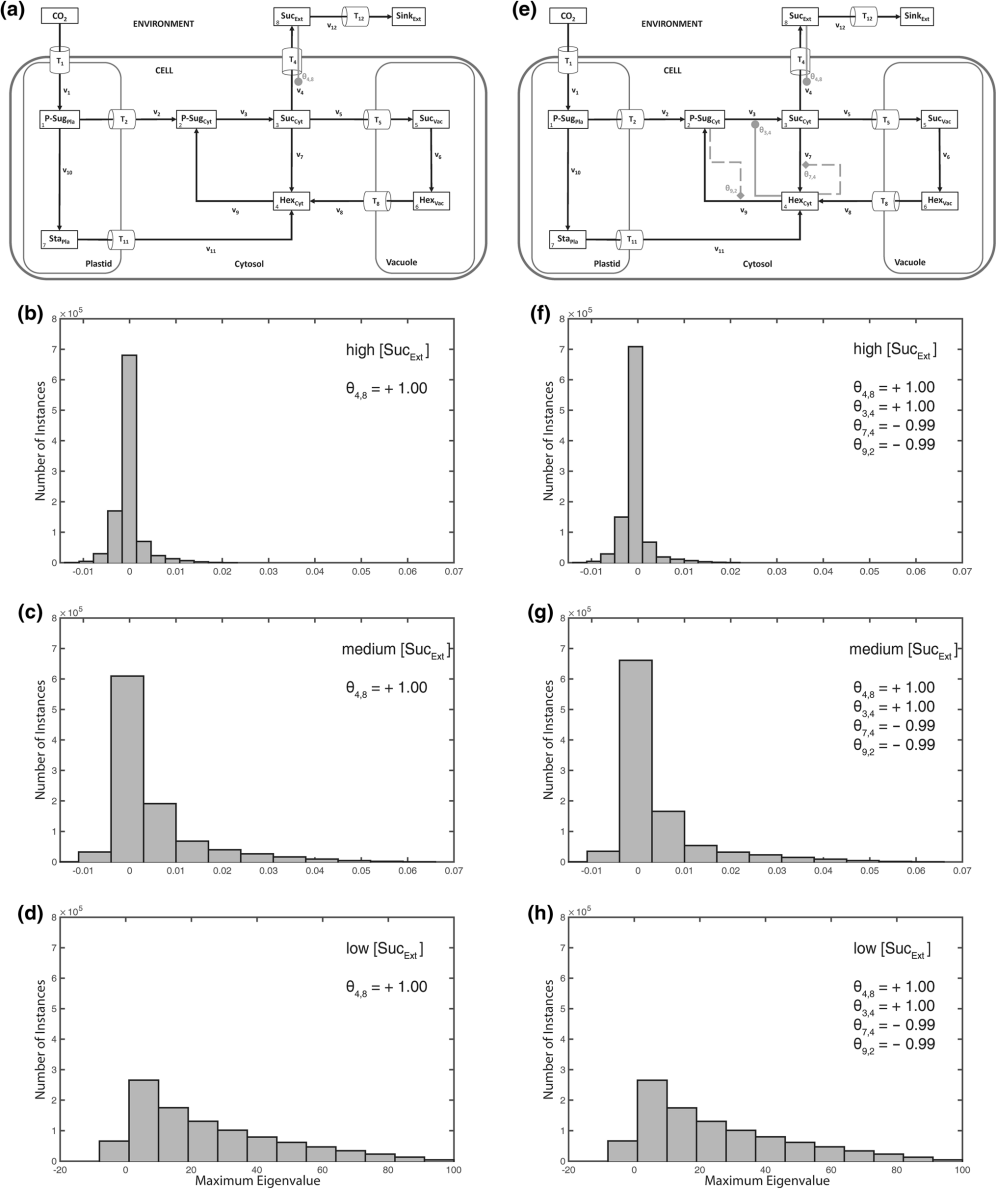
Activation of sucrose export by extracellular sucrose. Calculations for the shown model configuration (**a**) were performed 10^6^ times for high (**b**, **f**), medium (**c**, **g**), and low (**d**, **h**) concentration of extracellular/apoplastic sucrose concentrations. Steps of metabolic activation are indicated by *grey filled circles*, steps of inhibition are indicated by *grey filled diamonds* and *dashed lines*. Particular settings in the ***θ*** matrix are indicated within the *single histograms*. Further detailed information, e.g. about maximum values of the eigenvalue real part distribution, is provided in Supplementary Table S4

To further analyse the possible role and effect of an extracellular pool on the intracellular system stability, we combined the previously discussed metabolic scenarios (Figs. 2, 3) with an activation of the export reaction *v*_4_ (Suc_Cyt_ → Suc_Ext_) by extracellular sucrose (*θ*_4,8_; Fig. 4). In a biological context, such a hypothetical scenario might be interpreted as the result of a molecular signalling process which connects sink organs, e.g. root, with the source organs, e.g. leaves. The experimental and theoretical analysis of such sink-source interactions represents a challenging and difficult to assess research question in current plant biology which has been focused by numerous studies during the last decades (e.g. see Bancal and Soltani 2002; Brauner et al. 2014; Milne et al. 2013; Paul and Foyer 2001; Sonnewald and Willmitzer 1992; Tiessen and Padilla-Chacon 2012; Turgeon and Wolf 2009). In our stability analysis, the activation of sucrose export from the intracellular to the extracellular environment resulted in a destabilization of the intracellular metabolic homeostasis (Fig. 4a–d) which could not be stabilized by strong feedback-inhibitions of the cytosolic sugar metabolism (Fig. 4e–h). Yet, although instabilities occurred under all considered external sucrose concentrations, we detected an increase of maximum positive real parts of eigenvalues with lower external sucrose concentrations.

**Fig. 5:**
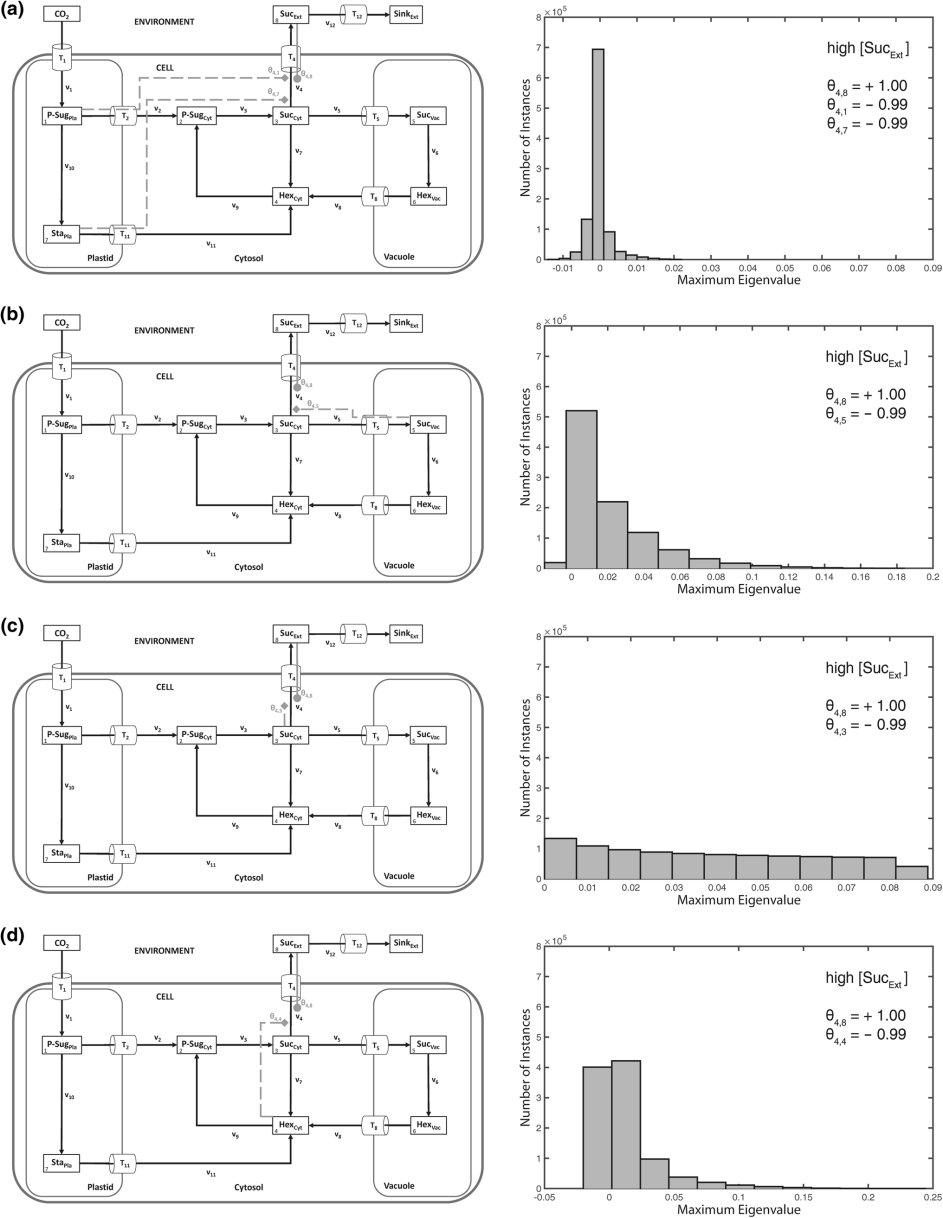
Differential regulatory stabilization of externally induced perturbation. Calculations for the shown model configurations (**a**–**d**) were performed 10^6^ times for high extracellular/apoplastic sucrose concentration. Distributions of resulting maximum eigenvalue real parts are shown in histograms. Steps of metabolic activation are indicated by *grey filled circles*, steps of inhibition are indicated by *grey filled diamonds* and *dashed lines*. Particular settings in the **θ** matrix are indicated within the *single histograms*. Further detailed information, e.g. about maximum values of the eigenvalue real part distribution, is provided in Supplementary Table S4

**Fig. 6:**
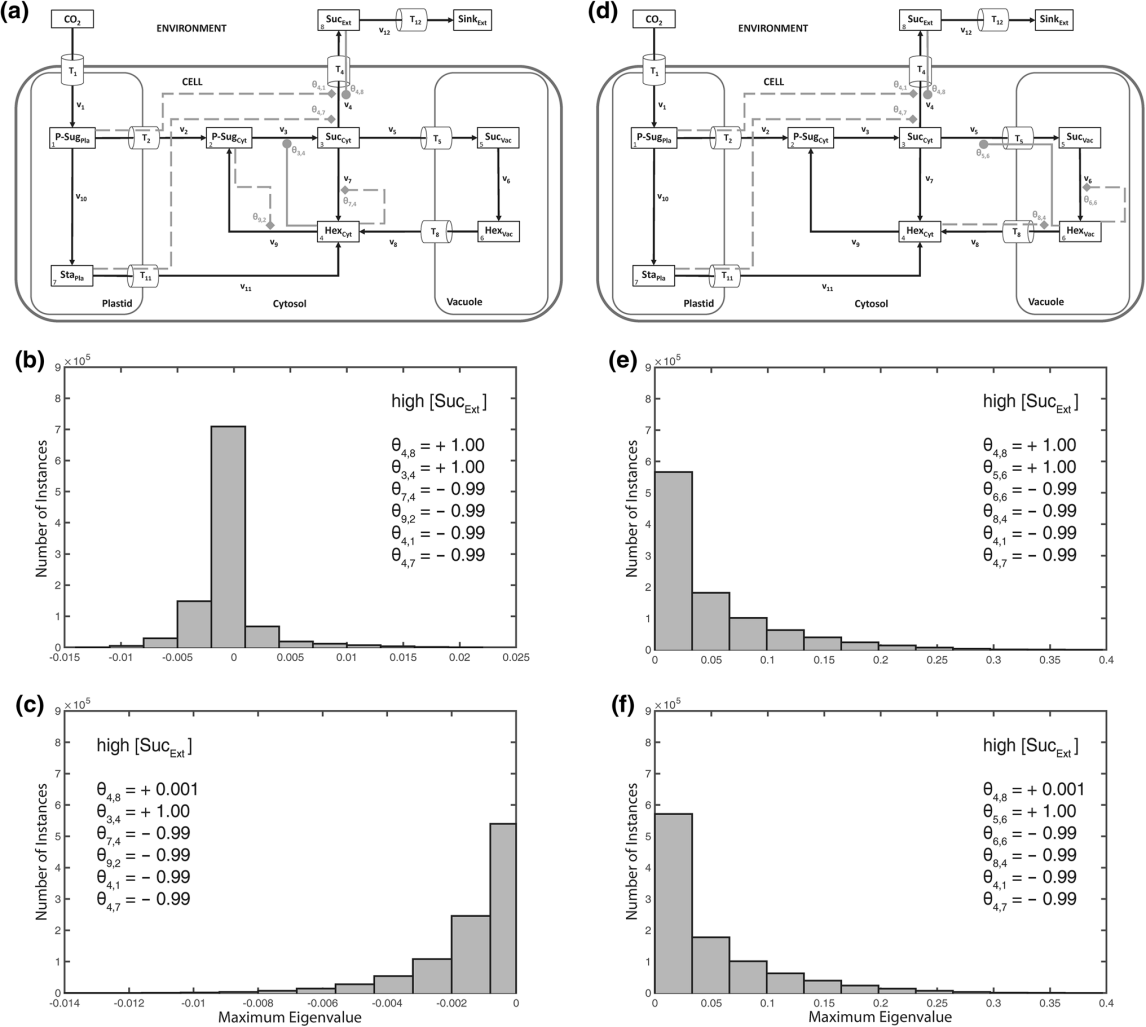
Differential stabilization output of cytosolic and vacuolar regulation. Calculations for the shown model configurations (**a**, **d**) were performed 10^6^ times for high extracellular/apoplastic sucrose concentration. Distributions of resulting maximum eigenvalue real parts are shown in *histograms* (**b**, **c**, **e**, **f**). Steps of metabolic activation are indicated by *grey filled circles*, steps of inhibition are indicated by *grey filled diamonds* and *dashed lines*. Particular settings in the ***θ*** matrix are indicated within the *single histograms*. Further detailed information, e.g. about maximum values of the eigenvalue real part distribution, is provided in Supplementary Table S4

**Fig. 7:**
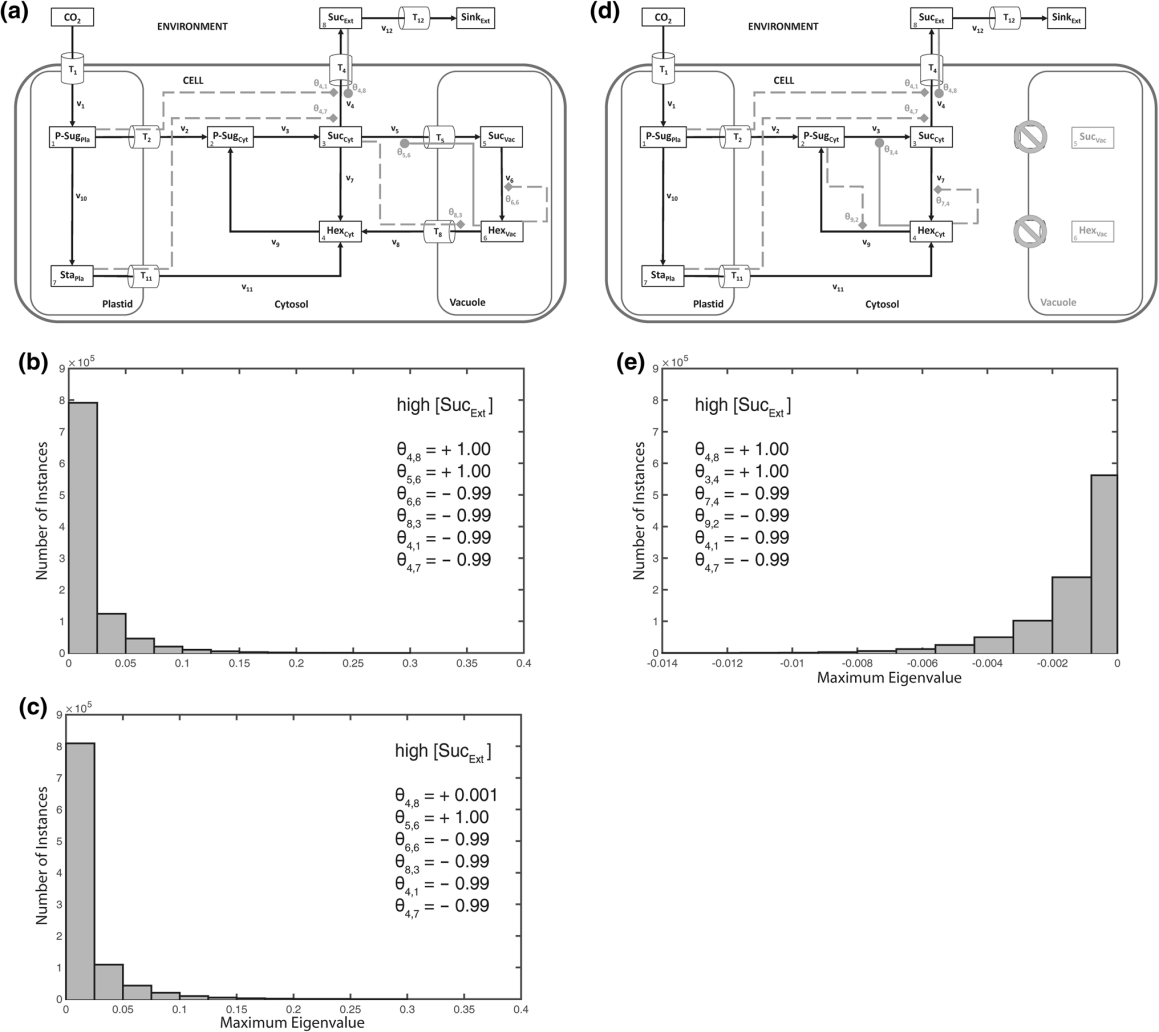
Contribution of vacuolar regulation to stabilization of a perturbed metabolic homeostasis. All calculations for the shown model configurations (**a**, **d**) were performed 10^6^ times for high extracellular/apoplastic sucrose concentration. Distributions of resulting maximum eigenvalue real parts are shown in histograms (**b**, **c**, **e**). Steps of metabolic activation are indicated by *grey filled circles*, steps of inhibition are indicated by *grey filled diamonds* and *dashed lines*. Particular settings in the **θ** matrix are indicated within the *single histograms*. Further detailed information, e.g. about maximum values of the eigenvalue real part distribution, is provided in Supplementary Table S4

Based on these observations, we explored various combinations of subcellular feedback-inhibition with regard to their capacity to re-stabilize the metabolic homeostasis after perturbation by a high external sucrose concentration (Fig. 5). In summary, neither a sole feedback-inhibition from plastidial (Fig. 5a) nor from vacuolar (Fig. 5b) nor from cytosolic (Fig. 5c, d) metabolic compounds could fully stabilize the system under all explored parameter constellations, i.e. elasticity variations. Further, also for the combination of plastidial and cytosolic regulatory instances, instable solutions were derived (Fig. 6a, b). Yet, in case of only a weak activation of sucrose export by extracellular sucrose (*θ*_4,8_ = 0.001), the combination of cytosolic and plastidial feedback-inhibition was found to yield a stable system (Fig. 6c). In contrast, a combination of plastidial with vacuolar (instead of cytosolic) regulatory instances was neither found to stabilize the system with strong nor weak activation of sucrose export by extracellular sucrose (Figs. 6d–f, 7a–c). In this context, we hypothesised that, following an extracellular perturbation, the vacuolar reactions of the model were negligible or even destabilizing the intracellular system. We proved this hypothesis by ignoring the vacuolar reaction cycle (Fig. 7d) which finally resulted in a stabilized system, even after strong activation by extracellular sucrose (Fig. 7e). This system stability was not persistent when vacuolar reactions were included (see Fig. 6a, b).

**Fig. 8:**
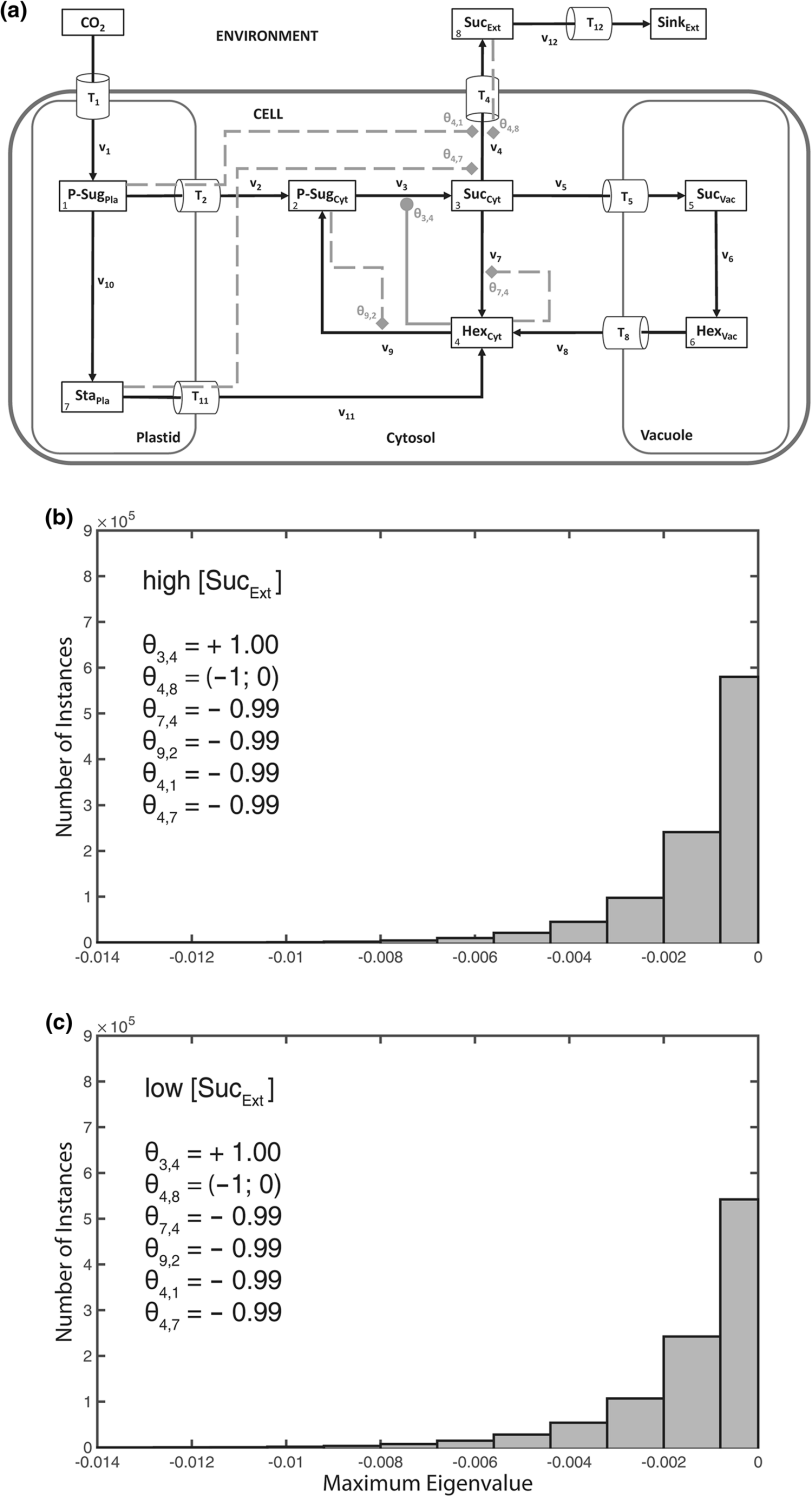
Stabilization output of external feedback inhibition. Calculations for the shown model configuration (**a**) were performed 10^6^ times for high (**b**) and low (**c**) extracellular/apoplastic sucrose concentration. Distributions of resulting maximum eigenvalue real parts are shown in both histograms. Steps of metabolic activation are indicated by *grey filled circles*, steps of inhibition are indicated by *grey filled diamonds* and *dashed lines*. Particular settings in the ***θ*** matrix are indicated within the *single histograms*. Further detailed information, e.g. about maximum values of the eigenvalue real part distribution, is provided in Supplementary Table S4

**Fig. 9:**
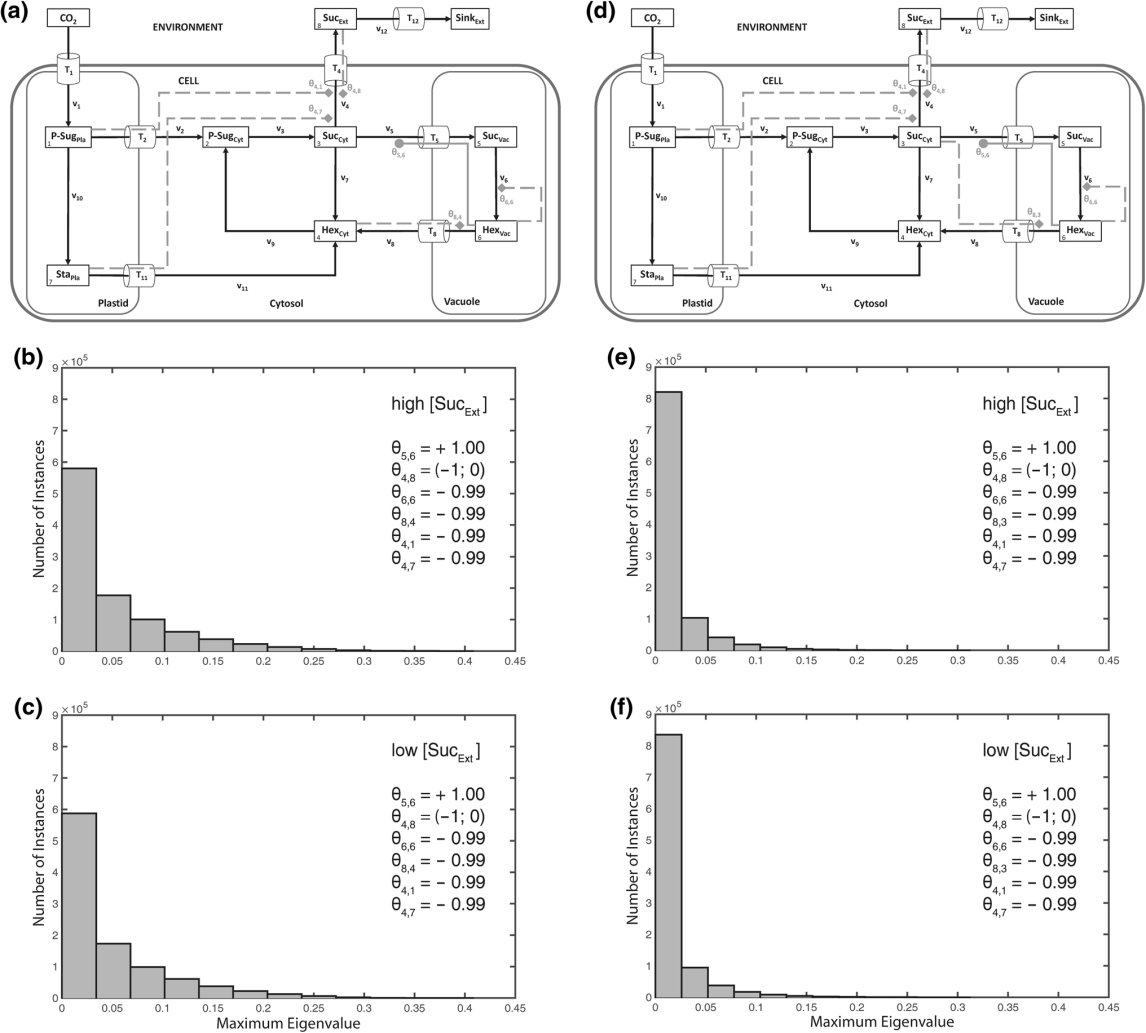
Destabilization by vacuolar regulation. Calculations for the shown model configurations (**a**, **d**) were performed 10^6^ times for high (**b**, **e**) and low (**c**, **f**) extracellular/apoplastic sucrose concentration. Distributions of resulting maximum eigenvalue real parts are shown in *histograms*. Steps of metabolic activation are indicated by *grey filled circles*, steps of inhibition are indicated by *grey filled diamonds* and *dashed lines*. Particular settings in the **θ** matrix are indicated within the *single histograms*. Further detailed information, e.g. about maximum values of the eigenvalue real part distribution, is provided in Supplementary Table S4

These observed outputs on steady state stability were related to the model assumption of an activation of sucrose export by extracellular sucrose, which, in a biological context, might be interpreted as a regulation of source metabolism by sink demand. To test a contrasting regulatory pattern, we replaced the activation term for extracellular sucrose on sucrose export by an inhibition term (*θ*_4,8_$\in$ (−1; 0); Fig. 8a). The combination of plastidial and cytosolic feedback-inhibitions resulted in a system stabilization irrespective of extracellular sucrose concentration (Fig. 8b, c). Even enhancing the previous observations, the replacement of cytosolic by vacuolar regulation yielded (purely) unstable solutions (Fig. 9) for both high and low extracellular sucrose concentrations.

## Discussion and conclusion

Metabolic reprogramming of biochemical networks due to environmental perturbations is a complex and multifaceted process. We have analysed a highly simplified, but still representative, biochemical network of the central carbohydrate metabolism in plant leaf cells with regard to its capability to respond and stabilize after a perturbation. Our results indicate that different subcellular organelles potentially contribute differentially to the reprogramming process, yielding a stable metabolic homeostasis only under certain preconditions. While it might not be surprising that different cellular organelles contribute differentially to metabolic regulation due to their different pH, volumes or proteomes (Ito et al. 2014; Joyard et al. 2010; Lunn 2007; Millar and Taylor 2014), the quantification of these contributions remains challenging. One example indicating the regulatory complexity on a subcellular and molecular level is the finding that vacuolar sugar carriers contribute differentially to metabolic reprogramming in different environmental conditions (Klemens et al. 2013). Klemens and co-workers showed that the metabolic consequences in the central carbohydrate metabolism, which were due to an increased activity of the vacuole-located carrier SWEET16, depended on the type of environmental stress. Based on their experimental observations the authors finally concluded that SWEET16 activity has to be tightly linked to developmental regulation in Arabidopsis under stress conditions (Klemens et al. 2013). Together with other classes of sugar transporters, SWEETs have also been suggested to be localized in the plasma membrane of phloem parenchyma cells and to play a central role in phloem loading (Chen 2014). While our proposed and analysed model of central carbohydrate metabolism does not account for different cell types, such as mesophyll and phloem parenchyma cells, but only differentiates between cellular metabolism and environment in general, our results might still be interpretable in this molecular context. One evident output of our stability analysis was the finding that feedback-inhibition originating from or directed to vacuolar carbohydrate metabolism had a less stabilizing effect on plastidial or cytosolic metabolism than we found it to occur vice versa. Although our approach is based on an abstract and highly simplified representation of these organelles, derived stability properties might still be representative for larger and more detailed networks as they arise from such small network modules which can be expected to occur frequently in large-scale networks. Hypothesising about a possible functional context of the observed destabilisation by the vacuolar part of the reaction network, we speculate that oscillatory processes, which might arise from instabilities, are possibly part of signal transduction mechanisms interconnecting carbohydrate metabolism of the vacuole with the cytosol. Previously, it has been discussed that oscillations in genetic or biochemical networks might represent a powerful mechanism to encode and transfer information both in time and space (Cheong and Levchenko 2010). This oscillatory information transfer might particularly play a role in vacuolar metabolism due to the diverse roles which it plays in plant leaf metabolism. First, it represents a cellular storage compartment for primary metabolites, but also for proteins or pigment molecules. Second, tonoplast monosaccharide transporters (TMTs) have been shown to be regulated by a mitogen-activated triple kinase (MAPKKK) which directly connects the vacuolar sugar metabolism to a central whole-cell signalling network affecting plant development, yield and stress tolerance (Neuhaus and Trentmann 2014; Wingenter et al. 2010). Third, in a previous study we have shown that vacuolar invertase activity, catalysing hydrolytic cleavage of vacuolar sucrose, affects whole plant carbon metabolism in *Arabidopsis thaliana* (Nägele et al. 2010). Although these examples comprise only a fraction of the in vivo metabolic and regulatory processes of vacuolar carbohydrate metabolism, it sums up to a complex regulatory picture which has to be tightly coupled to other cellular processes occurring in different compartments. Further, as sucrose represents the main transport sugar in the phloem (Lemoine et al. 2013), and, hence, links carbohydrate metabolism of source and sink tissue, the homeostasis of its intracellular levels and the concentration gradient across the plasma membrane are of central importance for carbon and energy metabolism of the whole plant. In contrast to carbohydrate metabolism of chloroplasts, which directly results in the pool of sugar phosphates being substrate for the whole-plant carbon homeostasis, vacuolar carbohydrate metabolism is attached to cytosolic metabolism which rather indirectly connects vacuolar metabolism to reactions of sugar biosynthesis and the transport of carbohydrates to sink organs. This might necessitate a differential regulatory strategy than in other compartments which could result in the observed characteristic stabilization pattern.

In conclusion, the presented findings indicate that, depending on extracellular fluctuations of metabolite concentrations, differential regulatory consequences might be necessary to efficiently stabilize intracellular networks. Particularly, in context of the very general question how sources interact with sinks, this indicates a highly dynamic interplay and allows for the speculation of the existence of multiple regulatory strategies which enable plants, and organisms in general, to respond to environmental fluctuations. Finally, it can be concluded that the diversity of molecular interactions between structural and regulatory elements of subcellular compartments contributes significantly to the stabilization of a cellular metabolic homeostasis.

## Electronic supplementary material

Below is the link to the electronic supplementary material.

Subcellular steady state concentrations applied for calculations (XLSX 9 kb)

Matrix **Λ** derived for the metabolic structure provided in Fig. 1 (PDF 201 kb)

Matrix ***θ*** derived for the presented metabolic structures (PDF 195 kb)

Detailed Information about eigenvalue real part distributions (XLSX 35 kb)

Matlab^®^ script for calculation of eigenvalues (ZIP 2 kb)

Histograms of maximum eigenvalue real parts with cytosolic activation and inhibition under varying steady state fluxes. Calculations for the shown model configuration (**a**) were performed 10^6^ times for low concentration of extracellular/apoplastic sucrose concentrations and for *F* = 0.5 (**b-e**) and *F* = 2 (**f-i**). Steps of metabolic activation are indicated by grey filled circles, steps of inhibition are indicated by grey filled diamonds and dashed lines. Particular settings in the **θ** matrix are indicated within the single histograms (TIFF 2445 kb)
