# Supplementary material for: Approximating the stabilization of cellular metabolism by compartmentalization
Source: Theory Biosci. 2016 Apr 5;135:73–87. doi: 10.1007/s12064-016-0225-y (PMC4870308; doi:10.1007/s12064-016-0225-y)
Supplement: Supplementary file 2 — Matrix Λ derived for the metabolic structure provided in Fig. 1 (PDF 201 kb) [file 12064_2016_225_MOESM2_ESM.pdf]

Supplemental File S2: Matrix **Λ**, derived for the metabolic structure provided in Figure 1.

$$
\Lambda = \begin{pmatrix}
\frac{\alpha F}{[PSuc_{Pla}]_0} & -\frac{\beta\alpha F}{[PSuc_{Pla}]_0} & 0 & 0 & 0 & 0 & 0 & 0 & 0 & -\frac{(1-\beta)\alpha F}{[PSuc_{Pla}]_0} & 0 & 0 \\
0 & \frac{\beta\alpha F}{[PSuc_{Cyt}]_0} & -\frac{F}{[PSuc_{Cyt}]_0} & 0 & 0 & 0 & 0 & 0 & \frac{F-\beta\alpha F}{[PSuc_{Cyt}]_0} & 0 & 0 & 0 \\
0 & 0 & \frac{F}{[Suc_{Cyt}]_0} & -\frac{\alpha F}{[Suc_{Cyt}]_0} & -\frac{\gamma(1-\alpha)F}{[Suc_{Cyt}]_0} & 0 & -\frac{(1-\gamma)(1-\alpha)F}{[Suc_{Cyt}]_0} & 0 & 0 & 0 & 0 & 0 \\
0 & 0 & 0 & 0 & 0 & 0 & \frac{(1-\gamma)(1-\alpha)F}{[Hex_{Cyt}]_0} & \frac{\gamma(1-\alpha)F}{[Hex_{Cyt}]_0} & -\frac{F-\beta\alpha F}{[Hex_{Cyt}]_0} & 0 & \frac{(1-\beta)\alpha F}{[Hex_{Cyt}]_0} & 0 \\
0 & 0 & 0 & 0 & \frac{\gamma(1-\alpha)F}{[Suc_{Vac}]_0} & -\frac{\gamma(1-\alpha)F}{[Suc_{Vac}]_0} & 0 & 0 & 0 & 0 & 0 & 0 \\
0 & 0 & 0 & 0 & 0 & \frac{\gamma(1-\alpha)F}{[Hex_{Vac}]_0} & 0 & -\frac{\gamma(1-\alpha)F}{[Hex_{Vac}]_0} & 0 & 0 & 0 & 0 \\
0 & 0 & 0 & 0 & 0 & 0 & 0 & 0 & 0 & \frac{(1-\beta)\alpha F}{[Sta_{Pla}]_0} & -\frac{(1-\beta)\alpha F}{[Sta_{Pla}]_0} & 0 \\
0 & 0 & 0 & \frac{\alpha F}{[Suc_{Ext}]_0} & 0 & 0 & 0 & 0 & 0 & 0 & 0 & -\frac{\alpha F}{[Suc_{Ext}]_0}
\end{pmatrix}
$$
