# Supplementary material for: Approximating the stabilization of cellular metabolism by compartmentalization
Source: Theory Biosci. 2016 Apr 5;135:73–87. doi: 10.1007/s12064-016-0225-y (PMC4870308; doi:10.1007/s12064-016-0225-y)
Supplement: Supplementary file 3 — Matrix θ derived for the presented metabolic structures (PDF 195 kb) [file 12064_2016_225_MOESM3_ESM.pdf]

Supplemental File S3: Matrix **θ**, derived for the metabolic structures presented in the main manuscript.

$$\boldsymbol{\theta} = \begin{pmatrix} 0 & 0 & 0 & 0 & 0 & 0 & 0 & 0 \\ \theta_{2,1} & 0 & 0 & 0 & 0 & 0 & 0 & 0 \\ 0 & \theta_{3,2} & 0 & \theta_{3,4} & 0 & 0 & 0 & 0 \\ \theta_{4,1} & \theta_{4,2} & \theta_{4,3} & \theta_{4,4} & \theta_{4,5} & \theta_{4,6} & \theta_{4,7} & \theta_{4,8} \\ 0 & 0 & \theta_{5,3} & 0 & 0 & \theta_{5,6} & 0 & 0 \\ 0 & 0 & 0 & 0 & \theta_{6,5} & \theta_{6,6} & 0 & 0 \\ 0 & 0 & \theta_{7,3} & \theta_{7,4} & 0 & 0 & 0 & 0 \\ 0 & 0 & \theta_{8,3} & \theta_{8,4} & 0 & \theta_{8,6} & 0 & 0 \\ 0 & \theta_{9,2} & 0 & \theta_{9,4} & 0 & 0 & 0 & 0 \\ \theta_{10,1} & 0 & 0 & 0 & 0 & 0 & 0 & 0 \\ 0 & 0 & 0 & 0 & 0 & 0 & \theta_{11,7} & 0 \\ 0 & 0 & 0 & 0 & 0 & 0 & 0 & \theta_{12,8} \end{pmatrix}$$
