# Supplementary figures and images for: Approximating the stabilization of cellular metabolism by compartmentalization
Source: Theory Biosci. 2016 Apr 5;135:73–87. doi: 10.1007/s12064-016-0225-y (PMC4870308; doi:10.1007/s12064-016-0225-y)

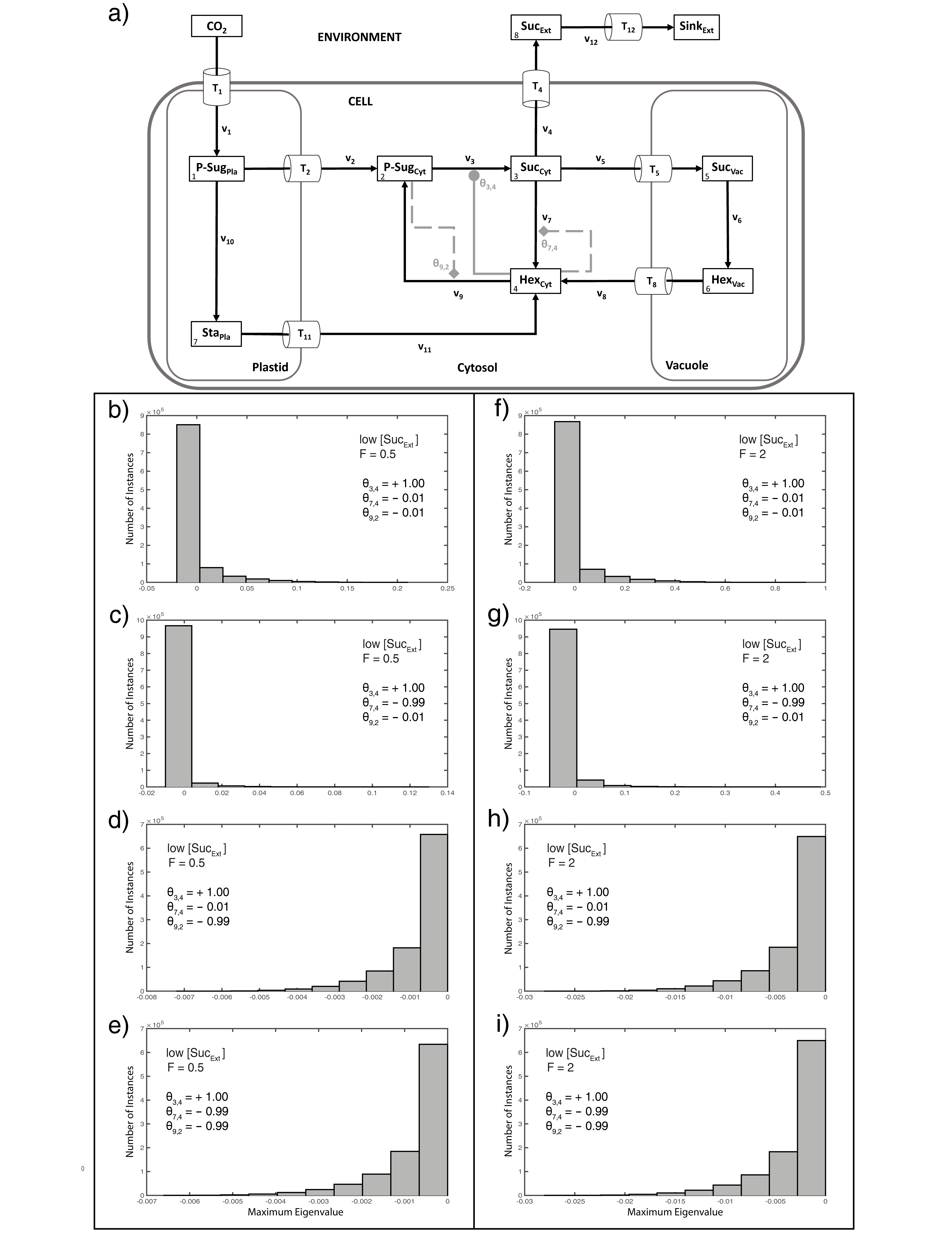

Supplement: Supplementary file 6 — Histograms of maximum eigenvalue real parts with cytosolic activation and inhibition under varying steady state fluxes. Calculations for the shown model configuration (a) were performed 106 times for low concentration of extracellular/apoplastic sucrose concentrations and for F = 0.5 (b-e) and F = 2 (f-i). Steps of metabolic activation are indicated by grey filled circles, steps of inhibition are indicated by grey filled diamonds and dashed lines. Particular settings in the θ matrix are indicated within the single histograms (TIFF 2445 kb) [file 12064_2016_225_MOESM6_ESM.tif]
